# Advances in the Chemical Properties and Functional Applications of Urushiol: From Traditional Lacquerware to Modern Materials

**DOI:** 10.3390/polym18091072

**Published:** 2026-04-29

**Authors:** Shanxiang Xu, Yutong Liu, Wenxuan Chen, Jiaxin Zhang, Xinyou Liu

**Affiliations:** 1 College of Furnishing and Industrial Design, Nanjing Forestry University, Nanjing 210037, China; xushanxiang@njfu.edu.cn (S.X.); 2311404209@njfu.edu.cn (Y.L.); wenxuanchen@njfu.edu.cn (W.C.); 3032751584@njfu.edu.cn (J.Z.); 2 Co-Innovation Center of Efficient Processing and Utilization of Forest Resources, Nanjing Forestry University, Nanjing 210037, China

**Keywords:** urushiol, bio-based polymers, dynamic covalent networks, self-healing materials, metal–phenolic coordination, cultural heritage conservation

## Abstract

Urushiol, the key component of natural lacquer, is emerging as a versatile bio-based phenolic platform for advanced polymer systems. Its unique catechol structure, combined with an unsaturated aliphatic side chain, provides multiple reactive sites, enabling diverse chemical pathways and tunable network architectures. This review presents a systematic analysis of urushiol-based materials within a “structure–reaction–property–application” framework. The intrinsic reactivity of urushiol, including oxidative polymerization, dynamic covalent bonding, and metal–phenolic coordination, is correlated with the formation of crosslinked networks exhibiting controllable mechanical properties, strong interfacial adhesion, and stimuli responsiveness. Recent advances in functional coatings, self-healing and reversible polymers, bioactive materials, and cultural heritage conservation are highlighted. Special emphasis is placed on dynamic network design and low-sensitization strategies to overcome limitations of traditional lacquer systems. Finally, key challenges and future directions toward controllable curing, structure–property relationships, and sustainable material design are discussed, positioning urushiol as a bridge between traditional materials and next-generation functional polymers.

## 1. Introduction

Natural lacquer, as one of the most representative traditional materials in East Asia, has been utilized for over 8000 years. It has been widely applied in furniture, utensils, and architectural decoration, and holds a significant position in cultural heritage [1,2,3]. Globally, natural lacquer is primarily sourced from trees such as *Rhus vernicifera* in China, *Toxicodendron vernicifluum* in Japan, and Toxicodendron succedaneum in Korea, with an annual harvest estimated at several thousand tons under sustainable tapping practices [1,2]. This production scale demonstrates the feasibility of urushiol as a renewable raw material for both industrial applications and cultural heritage conservation. From a materials science perspective, natural lacquer is a typical oil-in-water natural polymer system, primarily composed of urushiol (approximately 60–70%), water, polysaccharides, glycoproteins, and laccase [1,4]. Among these components, urushiol serves as the core active constituent and plays a decisive role in determining the overall properties of lacquer.

Structurally, urushiol consists of a catechol moiety and a C15–C17 unsaturated aliphatic side chain, exhibiting an amphiphilic characteristic of a “polar aromatic ring–nonpolar side chain.” This unique structure not only imparts excellent interfacial activity but also provides multiple reactive sites, including phenolic hydroxyl groups, active aromatic positions, and side-chain double bonds. Consequently, urushiol demonstrates high chemical tunability and versatility for functional material design [5,6]. Compared with other bio-based phenolics, such as lignin, tannins, and dopamine analogs, urushiol exhibits superior interface adhesion, controllable oxidative crosslinking, and dynamic network formation capabilities, making it particularly suitable for functional coatings and responsive polymer systems.

During the curing process, urushiol undergoes enzymatic oxidation catalyzed by laccase, generating semiquinone and o-quinone radicals. These intermediates further participate in radical coupling and side-chain crosslinking reactions, gradually forming a dense three-dimensional crosslinked network. This process can occur under humid and warm conditions or be initiated by ultraviolet irradiation [4,7]. The resulting lacquer films exhibit excellent durability, corrosion resistance, and gloss retention [8]. However, the highly reactive intermediates (e.g., o-quinones) produced during oxidation can undergo nucleophilic addition reactions with proteins, thereby triggering a typical type IV delayed hypersensitivity response. This intrinsic allergenicity limits the broader application of urushiol-based materials in modern materials science and biomedical fields [6,9].

In recent years, driven by the growing demand for green and sustainable materials, urushiol has attracted increasing attention as a renewable natural monomer for advanced functional materials. Owing to the presence of multiple reactive sites, urushiol is capable of undergoing diverse chemical transformations—such as oxidative polymerization, Michael addition, and metal–phenolic coordination—enabling the fabrication of functional material systems with tailorable architectures and performance [5]. Meanwhile, its potential functionalities in antibacterial activity, antioxidation, and interfacial bio-regulation have been progressively explored [10,11].

In the field of cultural heritage conservation, urushiol-based materials are not only integral components of traditional restoration systems but also face challenges such as aging sensitivity and material compatibility. For instance, environmental factors such as light exposure can induce cracking, loss of gloss, and structural degradation of lacquer films [2,3]. Therefore, a systematic understanding of the “structure–reaction–property” relationship of urushiol at the molecular level is of great importance for the development of novel functional materials and the advancement of conservation technologies.

**Figure 1:**
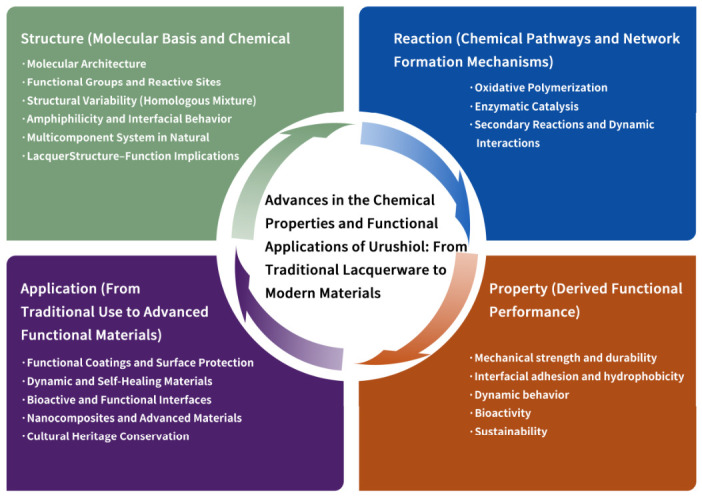
Schematic diagram of the overall structure of this review.

Based on these considerations, this review focuses on the chemical structure and reaction mechanisms of urushiol, and systematically summarizes its recent advances in functional coatings, dynamic polymer materials, bioactive materials, and cultural heritage conservation. Furthermore, future development trends, including hypoallergenic modification and intelligent material design, are discussed. The overall framework of this article is illustrated in Figure 1. This review is organized along the main line of “structure–reaction–property–application”: first, the molecular structure and chemical characteristics of urushiol are analyzed; subsequently, its reaction mechanisms and functional formation pathways are elucidated; then, its applications in coatings, dynamic materials, and biorelated fields are summarized; finally, future perspectives are proposed from the viewpoints of material design strategies and interdisciplinary integration, thereby establishing a systematic research framework bridging fundamental chemistry and practical applications.

## 2. Chemical Characteristics of Urushiol

### 2.1. Basic Structure and Composition

Urushiol is the principal active component of natural lacquer and fundamentally represents a class of natural phenolic compounds characterized by a catechol core structure linked to a long aliphatic side chain [5,12]. Its typical molecular structure consists of an aromatic ring bearing ortho-dihydroxyl groups and a C15–C17 side chain, which generally contains 0–3 unsaturated double bonds, forming a homologous mixture with varying degrees of unsaturation [12]. This structural feature, described as a “polar aromatic ring–nonpolar long chain,” endows urushiol with pronounced amphiphilicity: the aromatic ring exhibits high chemical reactivity, while the long aliphatic chain provides hydrophobicity and molecular flexibility, enabling the formation of dense and stable three-dimensional crosslinked networks during film formation [8].

**Figure 2:**
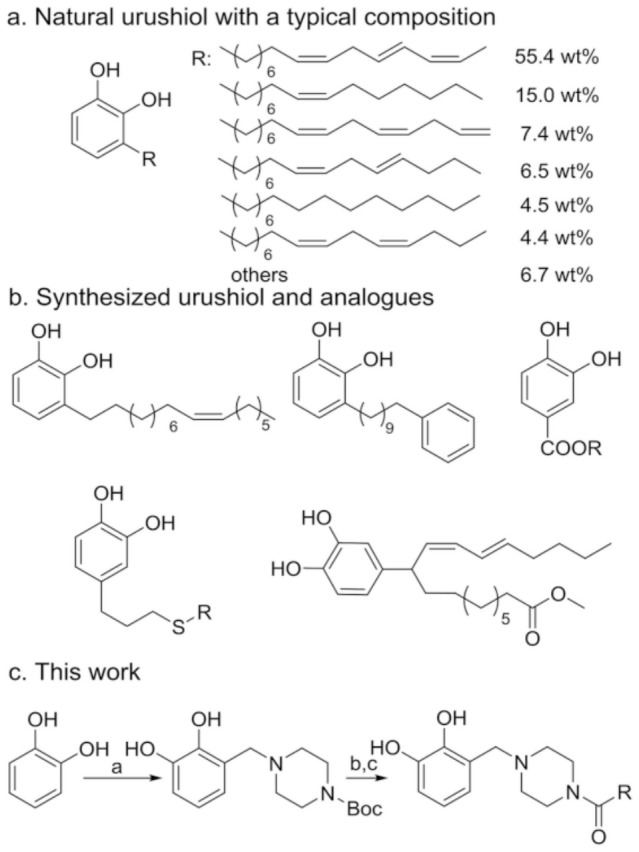
Schematic representation of urushiol molecular structures with varying side–chain unsaturation [14].

From the perspective of molecular composition, urushiol in natural lacquer is not a single compound but a mixture of structurally similar monomers, with variations primarily arising from differences in side-chain length and degree of unsaturation (Figure 2). For instance, triene-type urushiol, containing a higher number of double bonds, exhibits enhanced oxidative reactivity, which directly influences the curing rate and crosslinking density of lacquer films [7]. Furthermore, significant differences in urushiol composition exist among lacquer tree species, such as *Rhus vernicifera* and *Melanorrhoea usitata*, resulting in regional variations in material properties [13].

In natural raw lacquer systems, urushiol typically accounts for 60–70% of the total composition, while the remaining components include water (approximately 20–30%), polysaccharides, glycoproteins, and enzymes such as laccase and stellacyanin [4]. These non-urushiol components not only maintain the stability of the oil-in-water emulsion system but also play crucial roles during the curing process. For example, laccase catalyzes the oxidation of urushiol, whereas polysaccharides and proteins contribute to interfacial stabilization and facilitate electron transfer processes [4].

It is noteworthy that both the content and composition of urushiol are influenced by multiple factors, including tree species, geographical environment, tapping season, and processing methods. Generally, lacquer sap collected under warm and humid conditions contains a higher proportion of unsaturated urushiol, which is favorable for subsequent oxidative polymerization. In contrast, low-temperature or dry environments may suppress laccase activity, thereby reducing curing efficiency [13]. Additionally, urushiol may undergo pre-polymerization or oxidation during storage, further altering its molecular distribution and reactivity [4].

Overall, the structural characteristics of urushiol can be summarized as a “coupled system of a catechol reactive core and variable long-chain side groups,” with its composition exhibiting the “coexistence of multi-component distribution and environmental sensitivity”. This complexity in structure and composition provides a crucial foundation for subsequent multi-pathway chemical reactions and the development of functional materials.

### 2.2. Chemical Reactivity

#### 2.2.1. Oxidative Self-Polymerization

The most fundamental reaction pathway of urushiol is its oxidative self-crosslinking polymerization, in which molecular oxygen acts as the primary oxidant. During this process, the catechol moiety is oxidized into semiquinone or o-quinone radical intermediates under the influence of oxygen or external stimuli such as light and heat. These reactive intermediates subsequently undergo radical coupling reactions to form dimers, which further evolve into a three-dimensional crosslinked network structure [4,12].

From a kinetic perspective, the oxidative polymerization of urushiol can be described as a multi-step process involving sequential rate-determining stages, including (i) oxidation of phenolic hydroxyl groups to generate phenoxy radicals or quinone intermediates; (ii) radical coupling reactions forming oligomeric structures; and (iii) subsequent crosslinking through side-chain reactions. Among these, the initial oxidation step and radical coupling are generally considered rate-controlling processes, as they govern the generation and consumption of reactive intermediates [4,15].

**Figure 3:**
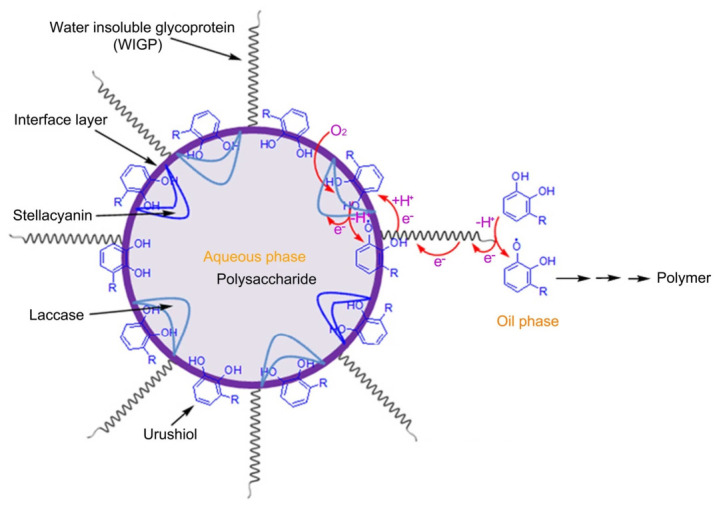
Schematic illustration of oxidative self-polymerization of urushiol at the oil–water interface [4].

In natural lacquer systems, this reaction exhibits pronounced interfacial regulation characteristics. Studies have shown that the oxidation of urushiol predominantly occurs at the water/oil interface. Radicals generated at the interface are transferred into the oil phase via electron transfer processes, thereby initiating bulk polymerization, as illustrated in Figure 3 [4]. Meanwhile, stellacyanin, a blue copper protein, plays a regulatory role by modulating radical concentration and suppressing uncontrolled rapid polymerization, resulting in a more uniform network structure.

The overall polymerization kinetics are strongly influenced by environmental and compositional factors. Temperature affects both enzymatic activity and radical mobility, thereby accelerating oxidation and crosslinking rates at elevated conditions. Humidity plays a crucial role in maintaining the stability of the water-in-oil emulsion and sustaining laccase activity, while oxygen concentration directly impacts the oxidation rate of catechol groups. In addition, enzyme concentration (e.g., laccase content) determines the rate of electron transfer and radical generation, thus influencing both local interfacial reactions and bulk network formation [4,15].

In addition to aromatic ring oxidation, the unsaturated double bonds in the aliphatic side chains can also participate in crosslinking reactions. Under ultraviolet irradiation or thermal activation, these double bonds undergo further radical-mediated reactions, enhancing both the crosslinking density and the mechanical properties of the resulting material [7]. Consequently, the oxidative polymerization of urushiol can be regarded as a kinetically coupled process involving interfacial-localized initiation and bulk network propagation, where the interplay between diffusion, radical concentration, and crosslinking reactions determines the final network structure and curing efficiency. Therefore, the polymerization of urushiol can be regarded as a synergistic process involving aromatic oxidative crosslinking and side-chain reactions.

#### 2.2.2. Enzymatic Catalysis Mechanism

**Figure 4:**
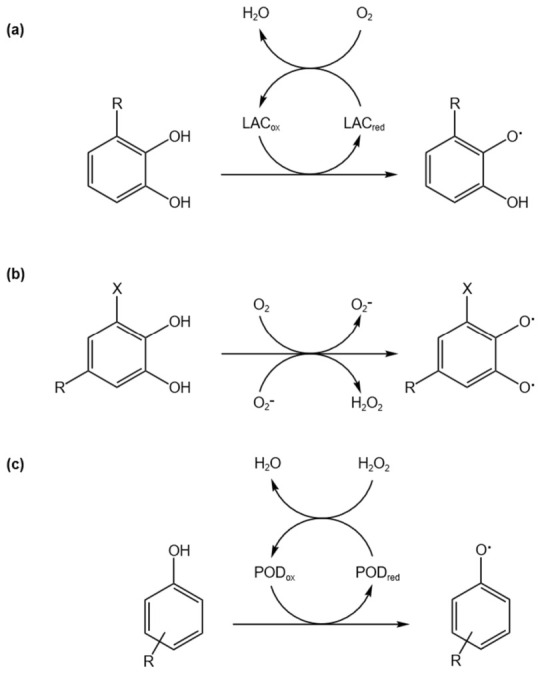
Mechanism of laccase-catalyzed oxidation of urushiol and subsequent radical coupling reactions (**a**) Laccase (LAC)-catalyzed oxidation of o-diphenols to phenoxy radicals with molecular oxygen (O_2_) as the terminal electron acceptor, producing water (H_2_O). (**b**) Laccase-mediated oxidation in the presence of mediators and reactive oxygen species (O_2_^−^, H_2_O_2_), leading to radical formation on substituted phenolic substrates. (**c**) Peroxidase (POD)-catalyzed oxidation of phenolic compounds using hydrogen peroxide (H_2_O_2_) as the oxidant to generate phenoxy radicals.

The curing of natural lacquers is fundamentally a typical enzymatic oxidative polymerization process, primarily driven by laccase. Laccase, a member of the multicopper oxidase family, catalyzes the oxidation of urushiol via a single-electron transfer mechanism, using molecular oxygen as the terminal electron acceptor to produce water, as illustrated in Figure 4 [15]. During this process, urushiol is initially oxidized to form phenoxy radicals, which subsequently undergo radical coupling reactions to generate primary polymeric structures.

From a kinetic standpoint, laccase-catalyzed oxidation significantly lowers the activation energy of phenolic oxidation and accelerates the formation of reactive intermediates, thereby shifting the rate-determining step from chemical oxidation to diffusion and interfacial electron transfer processes. This enzymatic pathway ensures a more controlled and sustained radical generation compared to purely thermal or photo-induced oxidation (15).

Glycoproteins present at the interfacial layer act as electron-transfer mediators, facilitating radical migration across different phases and thereby accelerating the overall polymerization process [4]. Additionally, stellacyanin, a blue copper protein, finely regulates radical concentration to suppress excessive oxidation, allowing precise control over both the polymerization rate and network uniformity. The coupling of enzyme catalysis with interfacial electron transfer further introduces spatial heterogeneity in reaction kinetics, where localized high-reactivity regions at the interface gradually propagate into the bulk phase, ultimately leading to the formation of a continuous three-dimensional network structure (4). This synergistic mechanism—“enzyme catalysis–electron transfer–interfacial regulation”—enables highly efficient and uniform curing even in the highly viscous lacquer system.

#### 2.2.3. Other Reaction Mechanisms

Beyond oxidative polymerization and enzymatic reactions, urushiol can participate in multiple chemical pathways that contribute to material construction and functional regulation. Firstly, the electrophilic o-quinone structures generated via oxidation readily undergo Michael addition or Schiff base reactions with thiol- or amino-containing molecules, forming stable covalent networks. This mechanism plays a crucial role in both material crosslinking and bio-interactions [5,16].

Secondly, the catechol moiety of urushiol can coordinate with metal ions such as Fe^3+^ or Cu^2+^, forming dynamic or multivalent crosslinked networks. Such metal–phenolic coordination not only significantly enhances mechanical performance but also lowers curing temperature and imparts reversible characteristics to the material [17].

Additionally, the phenolic hydroxyl groups of urushiol can be functionalized via esterification or etherification reactions with groups such as acrylates or isocyanates, thereby expanding its applicability in UV-curable materials, polyurethanes, and polymer composites [18,19]. Collectively, the multi-pathway reactivity of urushiol not only supports the construction of stable crosslinked networks but also provides a foundational basis for its extension to dynamic and functional material systems.

### 2.3. Characterization Techniques

To achieve a comprehensive understanding of the structure–reaction–performance relationship of urushiol-based materials, a range of advanced characterization techniques has been widely employed.

Spectroscopic methods, including Fourier transform infrared spectroscopy (FTIR), Raman spectroscopy, and UV–vis spectroscopy, are essential tools for monitoring the chemical evolution during oxidative polymerization. FTIR can be used to track the consumption of phenolic hydroxyl groups and the formation of quinone structures, while Raman spectroscopy provides complementary information on aromatic ring vibrations and conjugated structures. UV–vis spectroscopy is particularly useful for detecting quinone intermediates and evaluating the progression of oxidation reactions. These techniques collectively enable real-time or quasi-real-time analysis of the oxidative polymerization process.

Morphological characterization techniques such as scanning electron microscopy (SEM), transmission electron microscopy (TEM), and atomic force microscopy (AFM) provide critical insights into the micro- and nanoscale structure of urushiol-based coatings and composites. These methods are especially valuable for assessing surface roughness, phase distribution, and the formation of hierarchical or interfacial structures, which are closely related to barrier properties and mechanical performance.

Mechanical characterization methods, including dynamic mechanical analysis (DMA) and nanoindentation, are employed to evaluate the viscoelastic behavior, stiffness, hardness, and crosslinking density of the polymer network. In particular, DMA can be used to determine the glass transition temperature (Tg) and storage modulus, which are directly correlated with network structure and curing degree. Nanoindentation enables localized mechanical property measurements, providing insight into heterogeneity within coatings or composite systems.

Chemical composition and surface analysis techniques such as X-ray photoelectron spectroscopy (XPS) and nuclear magnetic resonance (NMR) spectroscopy play a crucial role in elucidating the bonding environment and molecular structure. XPS is particularly useful for analyzing surface chemical states and confirming metal–catechol coordination or oxidation states, while NMR provides detailed structural information on molecular modifications and polymer backbone evolution.

Overall, these characterization techniques are indispensable for systematically evaluating the oxidative polymerization process, crosslinking density, and functional performance of urushiol-based materials. By integrating multi-scale analytical approaches, it becomes possible to establish a clearer correlation between molecular structure, reaction pathways, and macroscopic properties.

## 3. Modern Functional Applications

### 3.1. Coatings and Surface Materials

As the core active component of natural lacquer, urushiol’s catechol moiety and unsaturated long-chain side groups confer exceptional oxidative crosslinking ability, enabling the formation of dense three-dimensional networks under air oxidation and enzymatic catalysis [20]. This network structure underpins the outstanding water resistance, corrosion resistance, and aging stability of natural lacquer coatings and is the key reason why traditional lacquerware and furniture surfaces can remain stable for centuries. Building on this foundation, inorganic–organic hybrid network structures can further enhance the protective performance of urushiol-based coatings. For example, ultrathin urushiol–Ti films constructed via layer-by-layer (LBL) assembly form highly dense three-dimensional networks through coordination between phenolic hydroxyl groups and metal ions, achieving corrosion inhibition efficiencies exceeding 99% and maintaining structural integrity even under harsh environments such as NaCl solutions and acidic media [21]. This indicates that urushiol is not only suitable for conventional thick coatings but also holds potential for development into nanoscale protective interface materials.

In addition to academic research, urushiol-based coatings have also been explored in patented technologies and practical applications, highlighting their industrial relevance. For instance, several patents have reported urushiol-modified anticorrosion coatings for marine and ship protection, where the combination of catechol-based adhesion and dense crosslinked networks significantly improves resistance to seawater corrosion and biofouling. In the field of architectural restoration, urushiol-based formulations have been patented for protective coatings on historical buildings, providing enhanced weather resistance and compatibility with traditional substrates. Furthermore, in cultural heritage conservation, patented urushiol-based restoration materials have been developed for lacquerware and wooden artifacts, emphasizing reversible protection, strong interfacial adhesion, and long-term stability. These patented technologies demonstrate that urushiol has transitioned from a traditional material to a functional platform with real-world engineering applications, bridging the gap between laboratory-scale research and industrial implementation.

Moreover, the catechol moiety in urushiol exhibits excellent interfacial adhesion, acting as a critical “interface bridging unit” in functional coating design [22]. In anticorrosion coatings, urushiol forms dense barrier layers through metal coordination, self-polymerization, and crosslinking, effectively hindering the diffusion and penetration of corrosive agents [21]. Particularly in ultrathin coatings, molecular-level assembly combined with light- or oxidation-induced polymerization enables high-density structures, overcoming the traditional reliance on coating thickness and establishing a structure-dominated protection strategy. The long aliphatic side chains of urushiol also impart low surface energy, resulting in superior hydrophobicity and interfacial regulation [22]. For instance, three-dimensional porous materials modified with urushiol can achieve highly efficient oil–water separation, with adsorption capacities up to tens or hundreds of times their own weight while maintaining excellent cycling stability [22]. Similarly, in carbon-based interface modification, urushiol reacts with graphene oxide (GO) and undergoes in situ polymerization to form a three-dimensional polymeric framework, significantly enhancing mechanical performance (>20 kPa), hydrophobicity, and functional potential in oil–water separation and anticorrosion applications [23].

With the trend toward multi-scale structural control in material design, urushiol has evolved from a simple film-forming component to a versatile structural and interface-regulating unit in composite materials. For example, poly(urushiol borate) systems formed via borate crosslinking can create core–shell microspheres and further develop into Janus composite particles, enabling anisotropic interface regulation and offering new structural design strategies for functional coatings [24]. In nanoscale composites, urushiol stabilizes and disperses nanoparticles uniformly through multiple interactions, including metal coordination and π–π interactions, thereby enhancing structural stability and functional performance [25]. This evolution demonstrates that the role of urushiol in composite coatings has transitioned from a traditional “film-forming substance” to a “core unit for interface engineering and structural regulation”.

**Figure 5:**
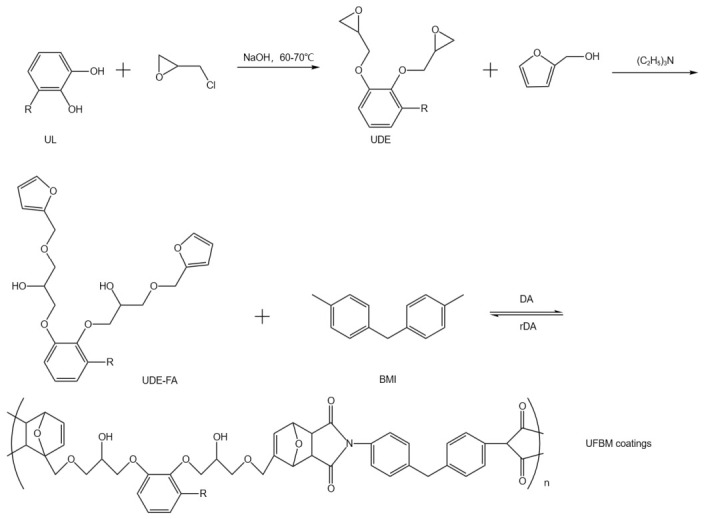
Construction Mechanism of Urushiol-Based Self-Healing Coatings via Diels–Alder Reactions.

In high-performance coating systems, dynamic covalent chemistries of urushiol have further advanced the development of self-healing coatings. Incorporation of dynamic covalent bonds, such as Diels–Alder (DA) linkages, enables thermally responsive reversible networks, allowing damaged coatings to recover their structure and functionality through heat-triggered bond exchange, as illustrated in Figure 5. Studies show that such urushiol-based self-healing coatings can achieve high repair efficiency (over 83%) while maintaining high hardness (5H) and excellent hydrophobicity (contact angle ~109°) [26]. This synergy of “high strength–high reversibility” provides a promising strategy for prolonging service life and improving material reliability.

In the context of sustainable material development, urushiol, as a renewable natural resource, exhibits significant potential in bio-based coating systems. By partially or fully replacing petroleum-based phenols in phenolic resin formulations, urushiol enables reduced dependence on non-renewable resources while forming highly crosslinked structures at relatively low curing temperatures (~100–113 °C), exhibiting excellent mechanical properties and thermal stability. Furthermore, urushiol-based materials demonstrate broad applicability across coatings, adhesives, and composites, underscoring their relevance in green manufacturing and sustainable material development, as illustrated in Table 1.

### 3.2. Reversible Materials and Dynamic Polymer Networks

With the continuous advancement of smart and sustainable materials, polymeric systems constructed via dynamic covalent bonds and reversible interactions have become a major research focus in materials science. Urushiol molecules, featuring both catechol moieties and unsaturated long-chain side groups, offer unique advantages in dynamic network construction. Their multiple reactive sites—including phenolic hydroxyls, aromatic reactive positions, and side-chain double bonds—not only support diverse crosslinking pathways but also enable reversible reactions that allow network restructuring, imparting key functions such as self-healing, reconfigurability, and environmental responsiveness.

This section centralizes all dynamic/reversible network mechanisms to avoid redundancy in later sections, establishing a unified framework of “dynamic network → self-healing → multi-scale applications.”

Mechanistically, the construction of urushiol dynamic networks relies on the formation of electrophilic o-quinone intermediates through oxidation, as well as reversible reactions involving the side-chain unsaturated bonds. Under oxidative conditions, urushiol is converted into o-quinone intermediates, which react with amino- or thiol-containing molecules via Michael addition or Schiff base reactions to form dynamic covalent networks [5]. These bonds can reversibly break and reform in response to external stimuli such as temperature or pH, endowing the material with structural adaptability. Additionally, catechol groups can coordinate with metal ions (e.g., Fe^3+^, Cu^2+^) to establish “coordination–covalent bond synergistic” dynamic networks. These systems maintain overall structural stability while enabling local reversible control, significantly enhancing mechanical performance and conferring stimuli-responsive properties, such as pH and ionic strength sensitivity [27]. Borate-based dynamic networks have also gained attention; for instance, poly(urushiol borate) systems exploit the dynamic exchange of B–O bonds to achieve tunable and reconfigurable structures [24]. Thus, the construction of urushiol dynamic networks fundamentally reflects the synergistic action of multiple types of dynamic bonds.

Beyond DA chemistry, multiple dynamic interactions—including disulfide exchange (S–S), hydrogen bonding, and π–π stacking—have been introduced into urushiol-based materials [28]. The cooperation of these weak interactions with covalent bonds imparts self-healing ability at both molecular and macroscopic scales, enhancing reliability and durability.

In composite systems, urushiol can act as a “dynamic connecting unit,” achieving structural reorganization and performance recovery through reversible crosslinking and interfacial interactions. For example, in graphene composites, urushiol forms dynamic crosslinks and interfacial interactions that maintain stable performance after multiple cycles [23]. Similarly, in Fe_3_O_4_–graphene catalytic systems, urushiol stabilizes nanoparticles and participates in interfacial reactions, improving cycling stability and structural tunability [25]. These studies indicate that urushiol serves not only as a building block for dynamic networks but also as a bridge connecting functional nanostructures to macroscopic material performance.

Overall, urushiol-based dynamic materials establish a clear “dynamic network → self-healing behavior → multi-scale functional applications” pathway, representing a typical paradigm of multi-pathway reaction coupling and multi-scale structural control. Their core advantage lies in balancing stability and reversibility through synergistic regulation of multiple dynamic bonds, both covalent and non-covalent. This feature endows them with broad application potential in self-healing coatings, smart responsive materials, and sustainable polymeric systems.

### 3.3. Bioactivity and Biomedical Potential

As a representative naturally occurring catechol compound, urushiol exhibits notable bioactivity in addition to its applications in traditional coatings and functional materials. Its molecular structure, comprising catechol groups and unsaturated long-chain side chains, confers significant potential for antibacterial, antioxidant, and interfacial bio-regulation functionalities. However, the intrinsic sensitization risk of urushiol limits its direct use in biomedical applications. Consequently, balancing bioactivity with toxicity control has become a central challenge in urushiol-based biomaterial research.

Mechanistically, urushiol and its derivatives demonstrate broad-spectrum antimicrobial activity, largely resulting from the synergistic effects of their phenolic structure and oxidative reactivity. The antibacterial mechanism can be described as a multi-pathway process: (i) the hydrophobic long-chain side groups insert into bacterial membranes, disrupting the lipid bilayer integrity and causing leakage of intracellular contents; (ii) quinone and radical intermediates generated during oxidation induce reactive oxygen species (ROS) formation, leading to oxidative damage of proteins and nucleic acids; and (iii) the electrophilic o-quinone structures can covalently bind to thiol or amino groups in intracellular proteins, inhibiting critical enzymatic activities [16]. These synergistic effects confer significant inhibitory activity against Gram-positive bacteria and certain fungi, and under specific conditions, potential antiviral activity has also been observed [10]. Consequently, urushiol holds great promise for antibacterial coatings and functional biomedical surfaces.

Owing to its antibacterial properties and strong interfacial adhesion, urushiol also demonstrates potential in food and medical surface engineering. In food packaging, urushiol-based coatings serve as natural antimicrobial barriers, using hydrophobicity and dense network structures to inhibit water and oxygen transmission while suppressing microbial growth, thereby extending shelf life and improving safety. In medical settings, urushiol can be employed to construct antibacterial functional surfaces for devices, high-touch interfaces, and surgical instruments. When combined with polymeric or inorganic materials, it forms stable coatings with long-term antimicrobial performance, reducing hospital-acquired infection risks. Notably, the catechol moiety of urushiol is structurally analogous to the DOPA residues in mussel adhesive proteins, providing excellent adhesion to diverse substrates and forming a molecular basis for biointerface engineering and tissue-adhesive materials design [27].

Despite these biofunctional advantages, urushiol’s potent sensitization remains a limiting factor. Immunologically, urushiol-induced allergic reactions are classified as typical Type IV delayed hypersensitivity. The core mechanism involves oxidation of urushiol to o-quinone intermediates, which then react with skin proteins via Michael addition or Schiff base formation to generate antigenic complexes, activating T-cell-mediated immune responses [9]. Additionally, volatile constituents of urushiol, such as terpenoids, may further exacerbate irritation through nonspecific inflammatory pathways, increasing sensitization risk.

To address these challenges, current strategies focus on molecular structure modification and materials design. Structural modifications, such as hydrogenation to reduce side-chain unsaturation or grafting polymers to mask phenolic hydroxyl groups, can effectively reduce reactivity toward biomolecules. Material design approaches, including covalent crosslinking or encapsulation, immobilize urushiol within polymeric networks, minimizing free monomer release and substantially lowering sensitization potential. Furthermore, nanocarrier- or composite-based controlled-release systems can regulate urushiol delivery, maintaining antimicrobial function while mitigating bio-stimulation. These strategies provide critical pathways for developing “low-sensitization, high-functionality” urushiol-based biomaterials, establishing a theoretical and technical foundation for applications in biomedical and food-contact materials.

Overall, urushiol in the biomaterials domain presents a dual nature of “bioactivity coexisting with biological risk.” Future development hinges on synergistic optimization of activity, safety, and stability through structural design and release modulation. This research direction not only holds significant implications for materials science but also offers a new paradigm for applying natural polymers in biomedical applications.

### 3.4. Applications in Cultural Heritage Conservation

As the core functional component of natural lacquer, urushiol has a long-standing history of application and distinct material advantages in the field of cultural heritage conservation. From traditional lacquerware restoration practices to modern functional coating design, its use is gradually transitioning from experience-driven craftsmanship toward mechanism-guided scientific material systems. Especially under the current conservation principles emphasizing “minimal intervention,” “reversibility,” and “material compatibility,” re-examining urushiol’s molecular structure and redesigning its functional features has become a key research direction linking traditional techniques with modern conservation technologies.

Traditionally, lacquer and its derivatives have been widely used to reinforce and restore lacquerware, wooden artifacts, and polychrome cultural relics. The primary advantages stem from the interface adaptability and structural stability of urushiol molecules. Specifically, the catechol moieties of urushiol can form stable interfacial interactions with wood, fibers, and mineral pigments via hydrogen bonding, π–π stacking, and metal coordination, enabling “compatible repair” between material layers. Concurrently, the oxidative polymerization of urushiol generates three-dimensional crosslinked networks, conferring excellent water resistance, corrosion resistance, and aging stability, which allow traditional lacquer layers to remain structurally stable over long-term burial or under complex environmental conditions [3]. Moreover, repeated applications of lacquer can produce gradient multi-layered systems, providing process adaptability in structural reinforcement, defect filling, and surface decoration. Nonetheless, these traditional systems exhibit limitations such as prolonged curing times, environmental dependency, and difficulty achieving reversibility—issues increasingly recognized under modern conservation principles and representing critical bottlenecks.

In this context, modified urushiol-based materials have been developed to construct controllable and reversible conservation systems. On the one hand, introducing dynamic covalent bonds (e.g., Diels–Alder linkages and Schiff-base bonds) or reversible coordination interactions allows the urushiol crosslinked network to dissociate and reassemble under specific conditions, achieving “repairable and removable” functionality [26]. For example, urushiol-based self-healing coatings constructed from dynamic covalent networks can undergo structural reorganization upon thermal stimulation, providing new strategies for localized repair. On the other hand, nanocomposite and multilayered designs enable localized controlled release and functional modulation of urushiol, mitigating the irreversibility issue of traditional materials. Interface-anchored urushiol on graphene or inorganic nanoparticles enhances material stability while maintaining performance over multiple cycles [23]. These studies demonstrate a shift from urushiol as a “permanent repair material” toward a “tunable functional material system”.

Furthermore, the development of dynamic materials and intelligent coatings offers novel technical pathways for heritage conservation. Urushiol-based materials, with multiple reactive sites and reversible reaction capabilities, possess unique advantages in constructing dynamic protective systems. In self-healing coating systems, urushiol-based dynamic networks can autonomously repair microcracks through bond exchange mechanisms, delaying material degradation—a particularly valuable feature for exposed wooden artifacts, lacquerware, and outdoor cultural heritage. At the interface engineering level, urushiol can form ultrathin protective layers via metal coordination and self-assembly (e.g., urushiol–Ti systems), providing effective protection without altering the artifact’s appearance, which aligns well with the “micro-intervention” principle in modern conservation. Additionally, urushiol’s incorporation into functional composite materials opens further conservation opportunities. In Fenton catalytic systems, for instance, urushiol not only serves as a structural connector but also participates in interfacial reactions, enhancing the material’s cyclic stability and functional efficiency, offering new strategies for pollutant degradation and environmental regulation of cultural relics [25].

Despite its promising applications, urushiol development in conservation faces multiple challenges. From a materials design perspective, there is an intrinsic conflict between durability and reversibility: highly crosslinked structures enhance long-term stability but reduce removability, making the construction of “controllable crosslinking–dynamic response” systems crucial. From a safety standpoint, urushiol’s sensitization limits its use in open environments, necessitating molecular modification or encapsulation strategies for risk mitigation. Regarding environmental adaptability, temperature and humidity variations significantly affect urushiol material performance, requiring robust environmental response and regulation mechanisms. Furthermore, current evaluation of urushiol materials in conservation largely relies on empirical judgment, lacking systematic quantitative assessment methods and standardized criteria. Future research should advance across multiple dimensions—“structural design, interface mechanisms, environmental response, and performance evaluation”—to achieve scientific and functional application of urushiol-based materials in cultural heritage preservation.

## 4. Development Strategies and Emerging Technologies of Urushiol-Based Materials

With the continuous advancement of materials science, urushiol has gradually evolved from a traditional natural coating precursor into a highly designable and tunable functional monomer platform. Its catechol core and unsaturated long-chain side groups provide multiple reactive sites, enabling hierarchical control from the molecular scale to macroscopic structures through molecular modification, interface engineering, and dynamic network construction. Consequently, urushiol-derived materials are increasingly developed according to a systematic logic of “structural design → reaction pathways → interface engineering → functional implementation.” This chapter systematically reviews recent representative studies, focusing on development strategies in chemical modification, nanocomposites, biomimetic interfaces, and functional material design.

### 4.1. Chemical Modification Strategies: From Natural Molecules to Functional Monomer Platforms

Urushiol contains both reactive phenolic hydroxyl groups and unsaturated side chains, allowing multiple chemical pathways for structural regulation and transformation from a natural monomer to a functional polymer unit. Among these strategies, epoxy ring-opening and esterification reactions to construct UV- or thermally curable prepolymers represent mature modification approaches. For instance, urushiol reacts with epoxy resin to prepare epoxy acrylate (UEA), which, combined with UV-curing, enables rapid curing and the construction of high-performance coatings, effectively overcoming the long curing time of traditional lacquer [29].

Condensation reactions also serve as an important pathway. Urushiol is condensed with formaldehyde to produce urushiol–formaldehyde resin (UFP), which could then be incorporated into composite coating systems to achieve synergistic improvement in mechanical and corrosion-resistant properties [30]. Meanwhile, the catechol structure of urushiol can participate in oxidative polymerization and free radical reactions, providing a molecular basis for constructing high-density crosslinked networks. This “natural monomer–synthetic polymer” transformation not only broadens the application scope of urushiol but also establishes it as a crucial building block in bio-based polymer systems.

### 4.2. Nanocomposites and Interface Engineering: Structural Reinforcement and Performance Enhancement

Nanocomposite strategies are key to improving the comprehensive performance of urushiol-based materials, focusing on interface structure regulation and filler dispersion optimization. Carbon-based nanomaterials, such as graphene and carbon nanotubes, can significantly enhance mechanical and barrier properties. For example, a GO/MWCNTs composite network formed multi-scale conductive and barrier structures, achieving a corrosion protection efficiency of up to 99.7% [30].

Two-dimensional nanomaterials, such as boron nitride nanosheets (BNNSs), have also attracted attention. Surface modification of BNNSs with urushiol (via interfacial interactions such as π–π stacking and hydrogen bonding) enhanced interfacial compatibility, significantly improving barrier and anti-corrosion performance [31]. Furthermore, bio-based nanomaterials such as nanocellulose have been employed to construct green composite systems, enabling simultaneous optimization of mechanical and electrochemical properties [29]. In porous materials, urushiol’s interfacial adhesion can modify three-dimensional substrates, producing superhydrophobic materials for oil–water separation [22].

In this section, the focus is placed on structural design strategies and interfacial engineering, while the underlying dynamic network mechanisms have been discussed in Section 3.2.

### 4.3. Bioinspired Interfaces and Adhesion Mechanisms: From Natural Inspiration to Engineering Applications

The catechol structure of urushiol closely resembles the DOPA residues in mussel adhesive proteins, providing a molecular basis for biomimetic interface material design. Waterborne adhesion mechanisms based on catechol-functionalized polyelectrolytes have been reported, in which solvent-induced phase separation and structural reconstruction enabled rapid (~25 s) and high-strength underwater adhesion, demonstrating a synergistic “electrostatic-phase separation–covalent crosslinking” mechanism [32].

Based on this, urushiol-based materials can achieve interfacial adhesion through multiple mechanisms, including metal coordination (e.g., with Ti or Fe^3+^), hydrogen bonding, and π–π interaction. This multi-mechanism synergy enables excellent adhesion under wet, rough, or porous conditions, providing a solid foundation for constructing low-surface-energy coatings [33].

Here, emphasis is placed on bioinspired interfacial design and adhesion performance, while reversible/dynamic network behavior is systematically discussed in Section 3.2.

### 4.4. Functional Material Design: Antibacterial, Antifouling, and Biointerface Regulation

In the functional direction, urushiol-based materials are evolving from structural materials to multifunctional material platforms. In antimicrobial systems, in situ reductions in silver ions by urushiol produce AgNPs/urushiol composites, achieving nearly 100% antibacterial efficiency and maintaining long-term stability through controlled release [34]. In biomedical applications, urushiol can crosslink with collagen, enhancing structural stability and dentin bonding strength while providing antimicrobial functionality [35].

For anti-fouling and marine applications, urushiol-based benzoxazine–copper polymers exhibit low surface energy, strong adhesion, and excellent antimicrobial and anti-fouling properties [33]. Antimicrobial coatings demonstrate dynamic evolution—initial high efficiency, mid-term decline, and long-term stability—closely related to crosslinking density and release behavior [36]. Additionally, silane modification or inorganic nanolayer intercalation can further improve coating weather resistance and anti-aging performance [37,38].

### 4.5. Insights from Cultural Heritage for Material Design Strategies

From the perspective of cultural heritage conservation, the development of urushiol-based materials demonstrates an evolutionary path from “traditional experience → modern modification → functional materials.” Archaeological evidence indicates that as early as ~8000 years ago, natural lacquer was used by early humans for coatings and bonding, with excellent water and corrosion resistance representing early high-performance materials [39]. Modern materials research has further revealed structure–performance relationships at the molecular level, providing guidance for heritage conservation: prioritize source consistency and interface compatibility, balance functionality and reversibility, and drive performance optimization through structural design. In the future, urushiol-based functional materials are expected to facilitate the transition of heritage conservation from simple “repair materials” to “intelligent protective materials.”

## 5. Challenges and Future Perspectives

Despite the broad application prospects of urushiol as a renewable and structurally designable natural polymer monomer in functional materials and heritage conservation, several critical challenges remain in both fundamental research and engineering implementation. From a technical standpoint, traditional urushiol-based materials rely on enzymatic catalysis and air oxidation for curing, which are highly sensitive to temperature, humidity, and oxygen concentration, leading to long curing cycles and limited process control [15]. The curing process involves oxidation of urushiol to quinone intermediates and subsequent radical coupling reactions, resulting in a complex and difficult-to-regulate reaction pathway [5]. Thermal curing can accelerate the process to some extent, but it may introduce heterogeneous crosslinking structures and performance discrepancies, further increasing system complexity [12].

From a biosafety perspective, the quinone intermediates generated during urushiol oxidation are highly reactive and can covalently bind to biological macromolecules, potentially triggering allergic or toxic responses [16]. Studies have shown that urushiol can induce typical contact dermatitis and immune reactions, with its sensitization remaining a major barrier to biomedical and open-environment applications [9]. For example, urushiol can activate sensory neurons via the IL-33/ST2 signaling pathway, eliciting pruritus and inflammation, highlighting the complexity of its allergenic mechanism [40].

At the material performance level, urushiol systems generally face the “high stability–low reversibility” dilemma. Highly crosslinked three-dimensional networks provide excellent durability and chemical stability but reduce removability and reprocessability [8]. In multiphase composite systems, such as heritage lacquer layers, differences in thermal expansion coefficients and interfacial compatibility among constituent materials can generate interfacial stress and microcracks, accelerating material degradation [36].

Importantly, in comparison with other bio-based phenolic materials such as lignin, tannin, and cardanol derivatives, urushiol demonstrates superior interfacial adhesion, faster oxidative network formation, and higher structural stability, while maintaining comparable or improved functional performance in coating and biomedical applications.

Therefore, urushiol uniquely integrates three-dimensional advantages of “scientific molecular design–sustainable material development–cultural heritage protection,” forming a coherent framework consistent with the graphical abstract of this work.

Furthermore, systematic understanding of structure–property relationships is still lacking. Although urushiol-based materials exhibit promising performance in functional applications—for instance, excellent adhesion and stability in conductive composites [41] and high cycling stability as lithium-ion battery binders [42]—quantitative models connecting molecular structure evolution with macroscopic properties remain undeveloped. At the interface level, the mechanisms of urushiol in multiphase systems are not fully elucidated; for example, semi-interpenetrating network structures in membrane materials can enhance performance, yet the associated interfacial transport and structural regulation mechanisms require further investigation [43].

Looking ahead, the development of urushiol materials is expected to follow a highly interdisciplinary trajectory, moving toward dynamic, intelligent, and sustainable systems. Reversible networks based on dynamic covalent bonds and metal coordination are emerging as promising research directions. For example, the introduction of metal ions (e.g., Fe^3+^) forming coordination bonds with catechol structures can enhance adhesion and mechanical strength while providing environmental responsiveness and partial reversibility [44]. Biomimetic multivalent networks and organic–inorganic hybrid strategies also offer new pathways for performance optimization, such as constructing multi-bonded networks to improve strength and durability [45] or employing biomimetic topologies to enhance interfacial adhesion [46].

Leveraging urushiol’s natural origin, its potential in green material systems is increasingly recognized. As a bio-based resource, urushiol holds significant value in replacing petroleum-derived materials, and its abundant plant sources and versatile chemical structure provide a solid foundation for functional material development [47]. Its long-term use in traditional lacquer arts further validates its stability and cultural significance as a natural polymer [3]. In heritage conservation, urushiol-based materials are gradually evolving from “traditional restorative coatings” to “smart protective materials.” Functional designs, including antimicrobial coatings, self-healing networks, and environmentally responsive protective systems, enable long-term surface protection and functional regulation. However, their long-term stability, biosafety, and material migration risks under complex environmental conditions still require systematic assessment [36].

Overall, the development of urushiol materials is at a critical stage of transition from empirical practice to science-driven design. Future research must advance along three dimensions—mechanistic understanding, material design, and application translation—by establishing quantitative structure–property models, developing low-sensitization modification strategies, and improving performance evaluation frameworks. Such efforts will promote the in-depth application and sustainable development of urushiol-based materials in high-performance functional materials and cultural heritage conservation.

## 6. Conclusions

This review systematically summarizes the chemical structural features, reaction mechanisms, and recent advances in the applications of urushiol in modern functional materials. As a typical natural catechol compound, urushiol is characterized by its catechol moiety and unsaturated long-chain side groups, providing multiple reactive sites and high molecular design flexibility. These features enable the construction of complex three-dimensional crosslinked networks through oxidative polymerization, enzymatic reactions, and various covalent and non-covalent interactions.

In terms of applications, urushiol has expanded from traditional natural lacquer coatings to functional coatings, dynamic polymeric materials, bioactive materials, and heritage conservation systems. Its excellent interfacial adhesion, antimicrobial performance, and structural tunability confer significant potential in corrosion-resistant coatings, self-healing materials, and biointerface engineering. Furthermore, materials based on dynamic covalent networks and biomimetic interface design offer new strategies to achieve reversibility and intelligent functionality.

Despite these advances, several key challenges remain, including limited curing efficiency, high sensitization potential, and the inherent trade-off between durability and reversibility. Addressing these challenges requires integrated approaches encompassing molecular structure modulation, dynamic network design, and mechanistic elucidation of interfacial interactions, aiming to establish a comprehensive “structure–reaction–performance–application” framework.

From a broader perspective, compared with other bio-based phenolic systems, urushiol exhibits a unique balance of interfacial activity, dynamic reconfigurability, and long-term structural stability, making it a distinctive candidate for high-performance sustainable materials.

Looking forward, urushiol serves as an important bridge connecting traditional materials with modern functional platforms. Future research should focus on the development of low-sensitization, high-performance materials, dynamic and self-healing systems, bio-based materials for sustainable manufacturing, and smart, controllable designs for heritage conservation. With the continued integration of materials science and cultural heritage protection, urushiol is poised to transition from a traditional craft material to a high-performance, intelligent functional material, providing new theoretical foundations and technological pathways for sustainable materials development and heritage preservation.

## Figures and Tables

**Table 1:** Quantitative comparison of urushiol with representative bio-based phenolic materials.

Material	Corrosion Resistance (Inhibition Efficiency/%)	Crosslinking Density (mol·cm^−3^)	Curing Time (h, 25–120 °C)	Antibacterial Efficiency (%)	Interfacial Adhesion Strength (MPa)	Typical Applications
Urushiol (natural lacquer system)	>99%	(2–6) × 10^−3^	6–24 (ambient oxidation)	85–99%	5–15	Protective coatings, cultural heritage conservation, biomedical interfaces
Catechol (dopamine-based systems)	85–95%	(1–3) × 10^−3^	12–48	70–90%	2–10	Bioadhesives, hydrogel coatings, tissue engineering
Tannic acid-based polymers	80–92%	(0.8–2) × 10^−3^	8–24	60–85%	1–6	Antibacterial films, food packaging, antioxidant coatings
Lignin-based phenolic resins	75–90%	(0.5–2.5) × 10^−3^	2–6 (thermal curing)	50–75%	2–8	Structural composites, adhesives, insulation materials
Cardanol-based resins (cashew phenol)	88–96%	(1–4) × 10^−3^	2–8 (thermal curing)	65–85%	3–12	Anticorrosion coatings, epoxy modification, adhesives

## Data Availability

No new data were created or analyzed in this study. Data sharing is not applicable to this article.
